# Pre-curettage cerclage in a viable triplet cervical pregnancy: A case report and review of literature

**DOI:** 10.18502/ijrm.v17i7.4864

**Published:** 2019-07-31

**Authors:** Atossa Mahdavi, Ashraf Aleyasin, Nazanin Sheibani

**Affiliations:** Department of Infertility, Shariati Hospital, Tehran University of Medical Sciences, Tehran, Iran.

**Keywords:** Cervical pregnancy, Cervical triplet pregnancy, Cervical cerclage, Methotrexate.

## Abstract

**Background:**

Cervical ectopic pregnancy (CEP) is a rare and dangerous form of ectopic pregnancy in which the blastocyst is installed within the endo-cervical canal. CEP diagnosis requires special awareness to evaluate patient precisely. Individualizing controversial medical and surgical management strategies is of importance in medical practice.

**Case:**

A 35-year-old nulliparous woman on her 9th week of pregnancy was referred to our hospital with vaginal bleeding preliminary misdiagnosed as aborting intrauterine pregnancy. Transvaginal ultrasound revealed an empty uterus and a viable triplet pregnancy just below the level of internal os. Cervical curettage after cerclage suture placement procedure removed conception tissues completely. Consequently, in the next few hours vaginal bleeding decreased to minimal amount and vital signs remained within normal limits and there was no hematocrit change. On follow-up day 32, serum B-HCG became negative.

**Conclusion:**

CEP diagnosis requires special attention and awareness to evaluate patient precisely along with skillful assessment of possible risk factors. Lifesaving treatment beside fertility preservation was successful with pre-curettage cerclage.

## 1. Introduction

Ectopic pregnancy (EP) is the implantation of blastocyst outside of the uterus cavity which accounts for approximately 6% of total pregnancies. Despite the modest incidence of EP, it remains the leading cause of hemorrhage, mortality, and morbidity during the first gestational trimester (1-3).

Cervical ectopic pregnancy (CEP) is a rare form of EP in which the blastocyst is installed within the endo-cervical canal. This type of pregnancy constitutes approximately 0.005% to 0.04% of pregnancies (4) and accounts for 3.7% of in-vitro-fertilization (IVF) ectopic pregnancies (5). The rich cervical vascularity, the incongruence of cervical biomechanics with gestational advancements, and the negligible incidence of CEP can significantly increase the potential of hemorrhage leading to mortality, morbidity, and infertility of the affected individual (6).

So, early diagnosis and appropriate conservative treatment especially in a woman's reproductive age is of great importance both for the patient and the physician. To the best of our knowledge, there is no previous report of viable triplet CEP in the literature. The objective of this case report is to introduce a rare case of triplet cervical pregnancy that infiltrated most of the cervix, and the diagnostic challenge the patient encountered and our management strategy for her fertility preservation.

## 2. Case Presentation

A 35-yearr-old pregnant woman G3A2 presented to Shariati Hospital, Tehran, Iran, with vaginal bleeding on March 30th, 2018. Her pregnancy was achieved by Intra Cytoplasmic Sperm Injection and Embryo Transfer performed on February 10th, 2018 in another IVF clinic in the country. From March 4th, she experienced an episode of heavy vaginal bleeding and then intermittent spotting and bleeding episodes without cramping pain. At the beginning, the ultrasound report was not significant, and the local health center diagnosed the problem as threatened abortion. Due to gradually aggravated bleeding, she was presented to our emergency department. The patient reported culmination an hour in advance of her admission. She did not report constitutional symptoms, nausea, vomiting, abdominal pain, flank pain, irritative and obstructive urinary symptoms, and odorous vaginal discharge supporting septic abortion. She had a three-year male factor primary infertility, unsuccessful IVF in 2015, and a two-year secondary infertility after two complete abortions without any dilation and curettage (D&C) procedure. She had a history of mild Mitral Valve prolapse and a small sub-serousal fibromyoma. The patient consumed routine gestational supplementary and denied tobacco and alcohol consumption. She did not experience pelvic inflammatory disease and previous pelvic surgery.

On physical examination, the patient was fully oriented and afebrile. Vital signs were stable. The abdomen was soft and non-tender. The patient's vaginal examination revealed a moderate hemorrhage with soft but closed cervical external os and normal-appearing vulva, vestibule, vaginal canal. She had minimal cervical motion tenderness. Other findings were unremarkable. The patient also revealed a sonogram report of March 29th, intrauterine pregnancy with signs of incomplete abortion from endo-cervix area. The patient also carried a normal cell count and β-HCG > 50,000 mIU/mL.

Upon arrival, her hemoglobin and hematocrit were 10.0 g/dL and 30%, respectively; serum β-HCG was 58399 mIU/mL. The transvaginal ultrasound scans surprisingly revealed a 10-mm endometrium but empty uterus and a live triplet pregnancy just below the level of internal os. Cervix was enlarged and the total diameter of the trophoblast was about 7 cm; the cervix was clearly infiltrated, and CEP was confirmed. Fetal heart rate was present in two of the three embryos. The patient was consulted about the diagnosis and available treatment strategies. She did not accept medical treatment with methotrexate. After getting the informed consent and under general anesthesia, cerclage sutures (without tightening) were placed around the cervical canal. Then, cervical arteries on 3 and 9 hours were clamped, and curettage was done, and conception tissues were removed completely. Then McDonald cerclage suture was tied tightly. During and after the procedure, the patient received oxytocin intravenously. Consequently, in the next few hours, vaginal bleeding decreased to minimal amount and vital signs remained within normal limits, and there was no hematocrit change. Next day, B-HCG dropped to 15,025 mIU/mL, and cerclage suture was removed five days later. On follow-up day 32, serum B-HCG became negative. On follow-up day 120, physical exam and ultrasound scan were normal. Fortunately, six months later, the patient came to my office reporting a new and normal singleton pregnancy.

## 3. Discussion

This article describes first, a patient with CEP preliminary misdiagnosed as aborting intrauterine pregnancy before presentation to our hospital and second, we managed to perform safely and completely the fertility preservation surgery on her. This article is written after getting patient's informed consent. There is a divergence of preferences among health care providers for CEP management which ranges from non-surgical methods to radical hysterectomy. However, a timely diagnosis increases the likelihood of implementing a more conservative method and retaining patient's fertility (5).

CEP is a rare form of EP with a very high risk of life-threatening hemorrhage and subsequent loss of fertility due to hysterectomy. Its risk factors are not fully understood (7), and the best medical or surgical treatment strategy should be individualized. Our patient reported two spontaneous abortions without previous uterine or cervical surgery and present pregnancy was achieved by assisted reproductive technology (ART). In 1994, Ginsburg* et al*. reported the first case of a triplet heterotopic gestation with a twin cervical pregnancy following IVF. The study underscored the surveillance of concurrent CEP in IVF conception (8).

The history of ART and painless bleeding beside closed cervical os, regular contours of three gestational sacs, and regular cardiac beats in two fetuses, no movement of the intracervical sac with gentle pressure to the cervix were enough diagnostic clues to differentiate CEP from incomplete abortion in our patient. Also, it was differentiated from scar pregnancy due to its location on the posterior wall of endocervical canal.

Controversial medical and surgical CEP management strategies, gestational age, hemodynamic status of the patient, fertility preservation request, available facilities, and physician expertise are important factors that should be considered for each patient. Different medical and surgical therapies are reported in medical publications. Single or multidose systemic methotrexate (MTX), intrasac injection of MTX or potassium chloride or absolute ethanol, or combinations of these therapies (9, 10), uterine artery embolization (UAE) (11), dilation and curettage with balloon insertion (12), hysteroscopy (13), and hysterectomy in uncontrolled hemorrhage or failed medical management are practiced in different centers. Case reports of cervical cerclage are successfully reported for the management of CEP especially in heterotopic pregnancies (14-20). When there are contraindications for medical therapy, or UAE, hysteroscopy are not available; cerclage techniques are good choices for some selected patients.

In this case report, we chronicled the stepwise sequence of clinical events that rotated a conventional incomplete abortion into the most recherché type of EP. However, the principal objective of this case report was to raise awareness on EP and to introduce our possibly avoidable stumbling blocks. First, in teaching hospitals with ob-gyn residents on the first line of treatment. Pragmatically this can imperil patient care by both insufficient clinical experience and false confidence. We suggest establishing comprehensive protocols and solid supervision. Also, an accurate and perceptive history taking can greatly assist identifying the risk factors associated with EP and strengthen the chance of timely diagnosis. Second, the patient's uncooperative attitude provoked a one-month deferment of proper diagnosis. This foregrounds the importance of education and psychiatric consult in patients with an enduring history of infertility. Last, the patient was admitted during the two-week Iranian Norouz spring holidays which accentuates the necessity of further caution on occasions susceptible to suboptimal accessibilities.

##  Conflict of Interest

There is no conflict of interest.
